# Pentoxifylline Attenuates Methionine- and Choline-Deficient-Diet-Induced Steatohepatitis by Suppressing TNF-****α**** Expression and Endoplasmic Reticulum Stress

**DOI:** 10.1155/2012/762565

**Published:** 2012-01-29

**Authors:** Min Kyung Chae, Sang Gyu Park, Sun-Ok Song, Eun Seok Kang, Bong Soo Cha, Hyun Chul Lee, Byung-Wan Lee

**Affiliations:** ^1^Division of Endocrinology and Metabolism, Department of Internal Medicine, Yonsei University College of Medicine, Seoul 120-752, Republic of Korea; ^2^College of Biomedical Sciences, CHA University, Seongnam 463-836, Republic of Korea; ^3^Advanced Institutes of Convergence Technology (AICT), Seoul National University, Suwon 443-270, Republic of Korea

## Abstract

*Background*. Pentoxifylline (PTX) anti-TNF properties are known to exert hepatoprotective effects in various liver injury models. The aim of this study was to investigate whether PTX has beneficial roles in the development of methionine- and choline-deficient-(MCD-) diet-induced NAFLD SD rats *in vivo* and TNF-**α**-induced Hep3B cells *in vitro*. *Methods*. SD Rats were classified according to diet (chow or MCD diet) and treatment (normal saline or PTX injection) over a period of 4 weeks: group I (chow + saline, *n* = 4), group II (chow + PTX), group III (MCD + saline), and group IV (MCD + PTX). Hep3B cells were treated with 100 ng/ml TNF-**α** (24 h) in the absence or presence of PTX (1 mM). *Results*. PTX attenuated MCD-diet-induced serum ALT levels and hepatic steatosis. In real-time PCR and western blotting analysis, PTX decreased MCD-diet-induced TNF-alpha mRNA expression and proapoptotic unfolded protein response by ER stress (GRP78, p-eIF2, ATF4, IRE1**α**, CHOP, and p-JNK activation) *in vivo*. PTX (1 mM) reduced TNF-**α**-induced activation of GRP78, p-eIF2, ATF4, IRE1**α**, and CHOP *in vitro*. *Conclusion*. PTX has beneficial roles in the development of MCD-diet-induced steatohepatitis through partial suppression of TNF-**α** and ER stress.

## 1. Introduction

Although the proposed theory [1, 2] of pathogenesis of nonalcoholic fatty liver disease (NAFLD) has been challenged, chronic inflammation plays a pivotal role in the development of fatty liver disease. TNF-*α* and IL-6, which are cytokines associated with inflammation, are found in higher levels in subjects with nonalcoholic steatohepatitis (NASH) than in those without simple NASH [3]. Consequently, a therapeutic strategy targeting TNF-*α* has been attempted to reduce fat accumulation and improve AST (aspartate aminotransferase) and ALT (alanine aminotransferases) in subjects with fatty liver disease [4–6]. Among the drugs used to suppress or inhibit TNF-*α* expression, pentoxifylline (PTX), a nonselective phosphodiesterase inhibitor reported to decrease TNF-*α* gene transcription as well as affect multiple steps in the cytokine pathway by direct or indirect inhibition of TNF-*α*, is currently used in the clinical field for treatment of cardiovascular disease [4, 7–9]. Several pilot studies have shown the beneficial effects of pentoxifylline on NAFLD and NASH [4, 5, 10]. The endoplasmic reticulum and oxidative stress of the initiation and progression of hepatic steatosis and inflammation have been implicated under metabolic stress conditions [11]. Recently, Zhang et al. [9] demonstrated ER stress-induced hepatic steatosis.

Based on these reports, we hypothesized that fat storage, triggered by cytokine-mediated inflammation, could be decreased through the alleviation of ER stress. Therefore, we investigated whether pentoxifylline has a beneficial role in methionine- and choline-deficient-(MCD-) diet-induced fatty liver disease in a rat animal model and examined molecular pathways related to ER stress.

## 2. Method

### 2.1. Cell Culture

Human Hep3B cells were cultured in Dulbecco's Modified Eagle Medium (DMEM, Welgene, Daegu, Republic of Korea) with 4.5 g/L glucose and 2 mM glutamine supplemented with 10% FBS, 1.5 g/L sodium bicarbonate, 100 IU/mL penicillin, and 100 ug/mL streptomycin. The medium was changed twice a week, and the cells were maintained in a 37°C incubator with 5%  CO_2_. The cells were subcultured when confluent (every 5~7 days) using trypsin (2.5 g/L) and EDTA (1 g/L).

For the western blot analysis, the cells were plated at 3 × 10^5^/well in 6-well plates and then treated with 100 ng/mL TNF-*α* (R&D system, Minneapolis, MN) in the absence or presence of 1 mM PTX (Sigma).

### 2.2. Animals and Experimental Protocol

Sprague Dawley (SD) rats (male, 220–280 g body weight) purchased from Orient Bio Inc. (Sungnam, Republic of Korea) were randomly divided into four groups (six rats per group) as follows. Group I: chow diet plus saline injection (once/day, i.p.); group II: chow diet plus PTX injection (50 mg/kg, once/day, i.p; PTX); group III: MCD diet plus saline injection (once/day, i.p.); group IV: MCD diet plus PTX injection (50 mg/kg, once/day, i.p.) for four weeks. Pentoxifylline (PTX) was purchased from Handock Pharmaceuticals (Seoul, Republic of Korea), and MCD diet was purchased from Dyets Inc. (Bethlehem, Pennsylvania). The rats were maintained at 60 ± 5% relative humidity and 22 ± 2°C, with a 12-hour light/dark cycle. Blood was obtained by cardiac puncture, and the livers were removed and weighed. The livers were fixed in 10% formalin or snap frozen in liquid nitrogen and then stored at −70°C for histologic analysis. All experimental procedures were performed under sterile conditions and approved by the Institutional Animal Care and Use Committee of Yonsei University College of Medicine.

### 2.3. Determination of Serum and Hepatic Biochemistry Levels

Aspartate aminotransferase (AST), alanine aminotransferase (ALT), total cholesterol (T-CHO), and triglyceride (TG) were quantified in serum using a commercial kit (Asanpharm Inc., Seoul, Republic of Korea). Frozen liver tissue was homogenized in 0.9% NaCl solution, and the homogenate was diluted to solution of 1 : 2 chloroform:methanol. The homogenate was mixed vigorously with vortex mixer and centrifuged at 1,000 rpm for 20 min. The upper phase was aspirated, and then the chloroform phase was used for the analysis of a variety of metabolite.

### 2.4. Histological Analysis

Fresh tissues were frozen immediately after each animal was sacrificed, and the tissue was placed in prelabeled base molds filled with embedding medium used for frozen tissue to ensure optimal cutting temperature (OCT). Routine frozen sections (7 *μ*m) were stained with oil-red O (Sigma, St Louis, MO). The paraffin-embedded sections were stained with hematoxylin & eosin (H-E) and Masson's trichrome. To evaluate hepatic steatosis, morphometric analysis was performed on two randomly selected fields (at 200 × magnification) of each animal section using an Olympus IX71 microscope with an Olympus DP70 camera (Olympus Optical Company, Tokyo, Japan).

### 2.5. Real-Time PCR

Total RNA from liver was isolated using Trizol Reagent (Invitrogen, Carlsbad, CA) according to the manufacturer's instructions. The hepatic mRNA levels of TNF-*α* were quantified by real-time PCR using the ABI PRISM 7500 sequence detection system (Applied Biosystems, Foster, CA) with TaqMan fluorogenic probes and primers for TNF-*α*: forward, 5′-AAT GGC CTC CCT CAT CAG TT-3′; reverse, 5′-CCA CTT GGT GGT TTG CTA CGA-3′. PCR reactions and analyses were obtained using Sequence Detector Software (Applied Biosystems, Foster, CA).

### 2.6. Western Blot

Homogenized liver tissues or Hep3B were lysed in lysis buffer (Intron Biotechnology, Sungnam, Republic of Korea) containing 50 mM Tris, pH 7.5, 150 mM NaCl, 1 mM EDTA, 1% Triton X-100, 1% sodium deoxycholate, 0.1% SDS, 1 *μ*M phenylmethylsulfonyl fluoride (PMSF), 5 *μ*g/mL aprotinin, and 5 *μ*g/mL leupeptin. The protein extracts were quantified using Bradford assay (Bio-Rad, Hercules, CA). The protein extracts were loaded into 10% SDS-PAGE, followed by transfer to nitrocellulose membrane (Bio-Rad). After blocking with 5% skim milk in 1XPBS, the membranes were incubated with each specific primary antibody, including GRP78 (Santa Cruz Biotechnology, Santa Cruz, CA), total and phospho-eIF2*α* (Cell Signaling Technology, Danvers, MA), ATF4 (Santa Cruz), ATF6 (ABNOVA, Taipei city, Taiwan), IRE1 (Santa Cruz), phospho-JNK (Cell signaling), CHOP, and *β*-actin (Santa Cruz), at 4°C overnight. The membranes were washed with TBS-T and incubated with peroxidase conjugated anti-rabbit IgG or anti-mouse IgG (Santa Cruz) for one hour at room temperature. The membrane was washed and incubated with detection solution (GE Healthcare, Buckinghamshire, NA, UK) for one minute and was then exposed to film. The signal intensity for each specific band on the western blots was quantified using National Institutes of Image J density analysis software (version 1.20).

### 2.7. Statistical Analysis

Statistical analysis was performed using PRISM (GraphPad Software Inc., San Diego, CA). Results are expressed as mean ± SD. Statistical significance was calculated using one-way analysis of variance (ANOVA) with a post hoc Bonferroni multiple comparison test to assess the differences between groups. Statistical significance was defined as the conventional *P* value of < 0.05.

## 3. Results

### 3.1. Effect of TNF-*α* and Pentoxifylline on Viability Hep3B Cells

**Figure 1:**
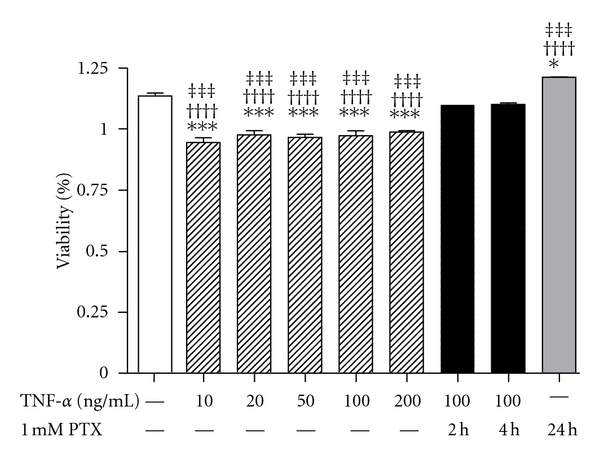
Viability of Hep3B cells after treatment with TNF-*α* and pentoxifylline. Hep3B cells exposed to 10, 20, 50, 100, and 200 ng/mL TNF-*α* for 24 hours showed a significant decrease in cell viability, assessed by MTT. Pretreatment with 1 mM pentoxifylline for 2 and 4 h prevented TNF-*α*-induced Hep3B toxicity. The bars represent percent cytotoxicity versus untreated controls. The experiments were performed four times under identical conditions. Results are shown as mean ± SEM. ***P < 0.001 *versus* untreated control cells; ^†††^ *P* < 0.001 *versus* 1 mM pentoxifylline for 2 h; ^‡‡‡^ *P* < 0.001 *versus* 1 mM pentoxifylline for 4 h.

Hep3B cells exposed to 10, 20, 50, 100, and 200 ng/mL TNF-*α* for 24 hours showed significantly decreased viability as assessed by MTT (1.00 ± 0.02 *versus* 0.83 ± 0.04, 0.86 ± 0.03, 0.85 ± 0.02, 0.86 ± 0.04, 0.87 ± 0.01, *P* < 0.001 for all). Compared to untreated controls, Hep3B cells treated with 1 mM PTX for 24 h showed statistically increased viability (1.00 ± 0.02 *versus* 1.07 ± 0.01, *P* < 0.05). Pretreatment with 1 mM pentoxifylline for 2 (0.97 ± 0.03) and 4 h (0.97 ± 0.01) significantly reduced TNF-*α*- induced Hep3B cell viability (*P* < 0.001 *versus* 10, 20, 50, 100, and 200 ng/mL TNF-*α*). Based on these results, we chose the concentrations of 100 ng/mL TNF-*α* and 1 mM PTX for this experiment (Figure 1).

### 3.2. Metabolic Effects of MCD Diet and Pentoxifylline on SD Rats

The amount of weight loss was different between rats given MCD plus saline (group III) and MCD plus PTX (group IV) for four weeks: −53.6 ± 9.2 g (19.6%) and –63.4 ± 10.2 g (23.4%) from their initial body weights. However, the difference was not significant. Such degree of weight loss is similar to previously reported data where rats were placed on MCD diets [1–3]. In contrast, rats in the control group gained a minimal amount of weight (4.6%) during the study period. Liver weight was not different among the four groups. The proportion of liver weight to body weight was similar between groups III and IV (Table 1).

**Figure 2:**
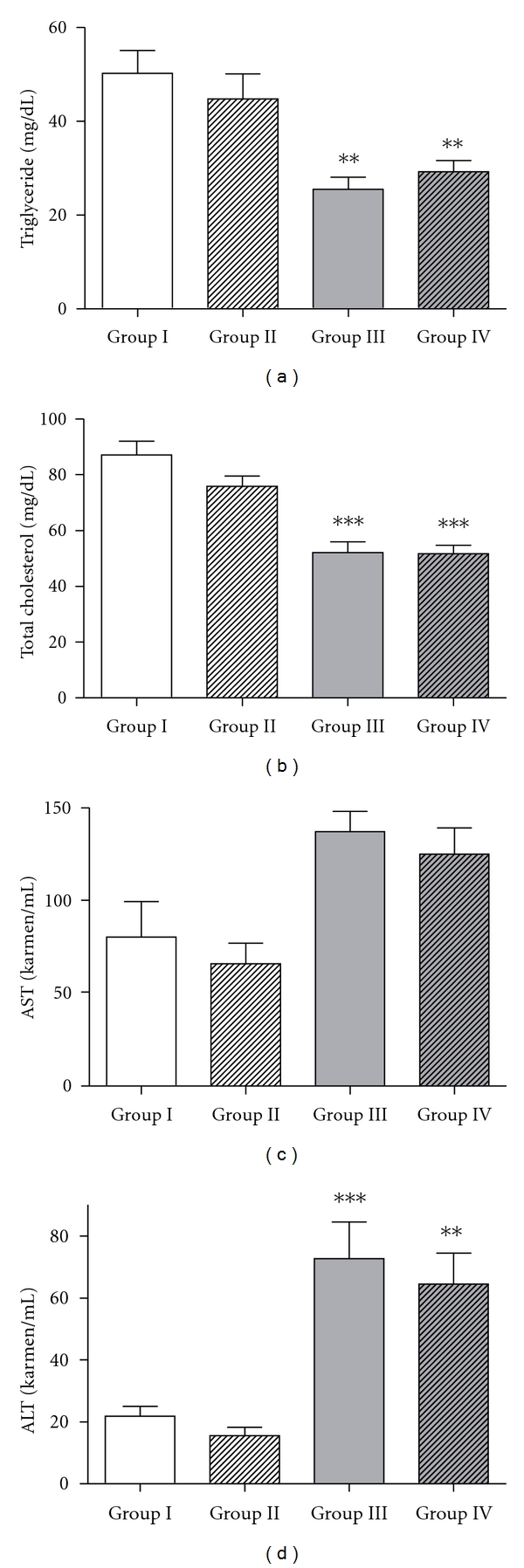
Serum chemistry values after 4 weeks of administration with an MCD diet and pentoxifylline. There was a significant decrease in serum triglyceride and total cholesterol in rats in the MCD plus saline (group III) and MCD plus PTX (group IV) groups relative to rats in control group (group I). A significant increase of ALT levels in groups III and IV was observed. Results are shown as mean ± SEM. **P < 0.01, ***P < 0.001 *versus* untreated control group.

The blood concentrations of TG, T-CHO, AST, and ALT were analyzed using serum. Rats in groups III and IV showed statistically decreased serum TG and T-CHO levels relative to rats in the control group. ALT levels were significantly increased in MCD-fed rats (groups III & IV) compared to the control groups, but there were no differences between groups III and IV (Figure 2).

### 3.3. Hepatic Effects of MCD Diet and Pentoxifylline on SD Rats

**Figure 3:**
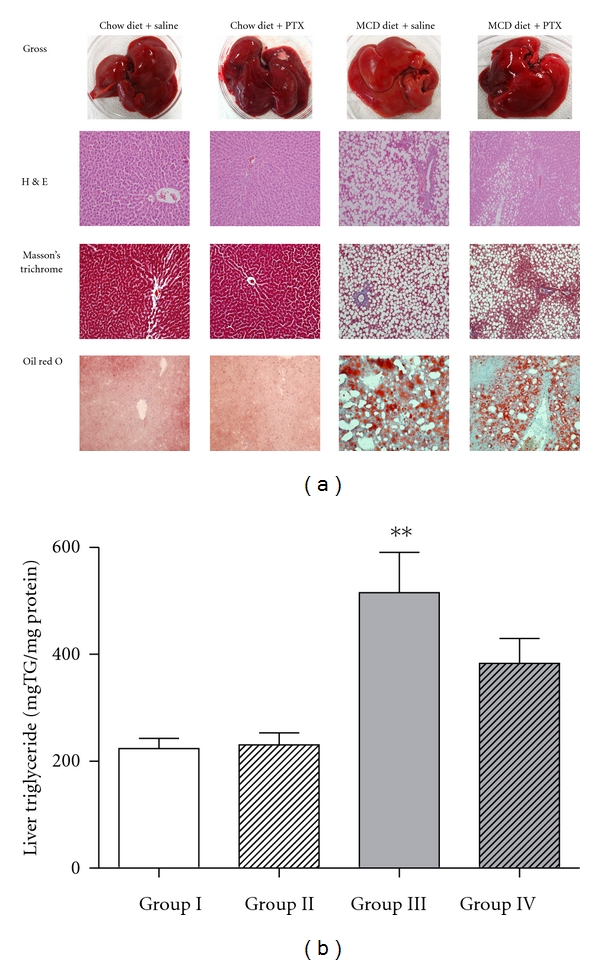
Hepatic histology and triglyceride content after 4 weeks of administration with an MCD diet and pentoxifylline. The liver sections were stained with H&E, Masson's trichrome, and oil-red O staining (×200). The liver of MCD-diet-fed rats showed yellowish markings. Rats administrated pentoxifylline showed the reduced yellow hepatic color and fat accumulation (a). Rats in groups III and IV have increased hepatic triglyceride content relative to rats in the control group. There was no statistical difference between groups III and IV (b). Results are shown as mean ± SEM. **P < 0.01 *versus* untreated control group.

Macroscopic and histological images of liver pathology were obtained. Liver sections were stained with H&E, Masson's trichrome, and oil-red O staining. In rats fed chow diet (groups I and II), there was no detectable fatty change in gross appearance and microscopic image as assessed by H&E and oil-red O staining. In contrast, the liver of MCD-diet-fed rats showed yellowish markings, typical of steatosis. Consistent with statistically increased serum ALT levels, extensive macrovesicular steatosis and minimal inflammation around perisinusoidal area were present in group III. In rats that were administrated pentoxifylline, the intensity of yellow color as well as fat accumulation was reduced. In addition to liver discoloration and fat accumulation, minimal perivenular fibrosis typically seen in NASH was found in MCD-fed rats (Figure 3(a)).

Rats in groups III and IV had increased hepatic triglyceride content relative to rats in the control group (515.1 ± 200.5, 375.7 ± 35.92 *versus* 223.6 ± 50.02 mg TG/mg protein), and significant increase was found in group III (*P* < 0.01). However, there were no statistical differences between groups III and IV (Figure 3(b)).

### 3.4. Effect of MCD-Diet-Induced Hepatic TNF-*α* Expression

**Figure 4:**
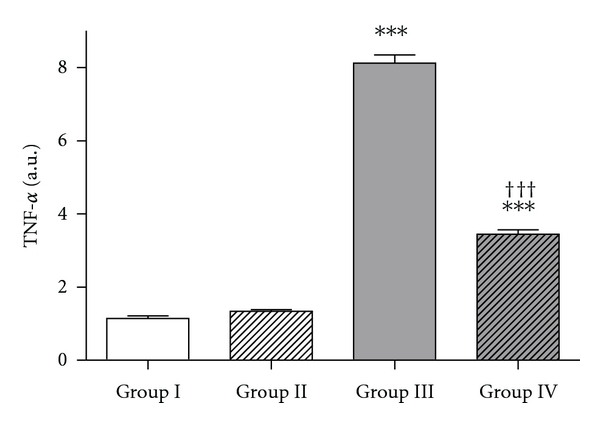
Hepatic TNF-*α* gene expression after 4 weeks of treatment with an MCD diet and pentoxifylline. There was a significant increase in liver TNF-*α* mRNA levels in rats fed MCD (groups III and IV). Administration of PTX in MCD diet rats significantly decreased the TNF-*α* mRNA expression. Results are shown as mean ± SEM. ***P < 0.001 *versus* untreated control group. ^†††^ *P* < 0.001: MCD plus saline *versus* MCD plus PTX group.

The hepatic TNF-*α* mRNAs from the four groups were measured using real-time PCR. Hepatic TNF-alpha gene expression was significantly increased in the MCD diet groups (groups III and IV) relative to the control group (1.14 ± 0.15 *versus* 8.12 ± 0.45, 3.45 ± 0.24, *P* < 0.001 for both). Administration of PTX (50 mg/kg, once/day, i.p) in MCD diet rats (group IV) significantly decreased TNF-*α* mRNA expression (*P* < 0.001) as compared to MCD-diet-fed rats in group III (Figure 4).

### 3.5. Effect of Pentoxifylline on TNF-*α*-Induced ER Stress in Hepatocytes and Hep3B Cells

Western blotting assay was performed to elucidate the hypothesis that downregulation of direct or indirect TNF-*α*-induced ER stress markers by pentoxifylline would attenuate hepatosteatosis.

**Figure 5:**
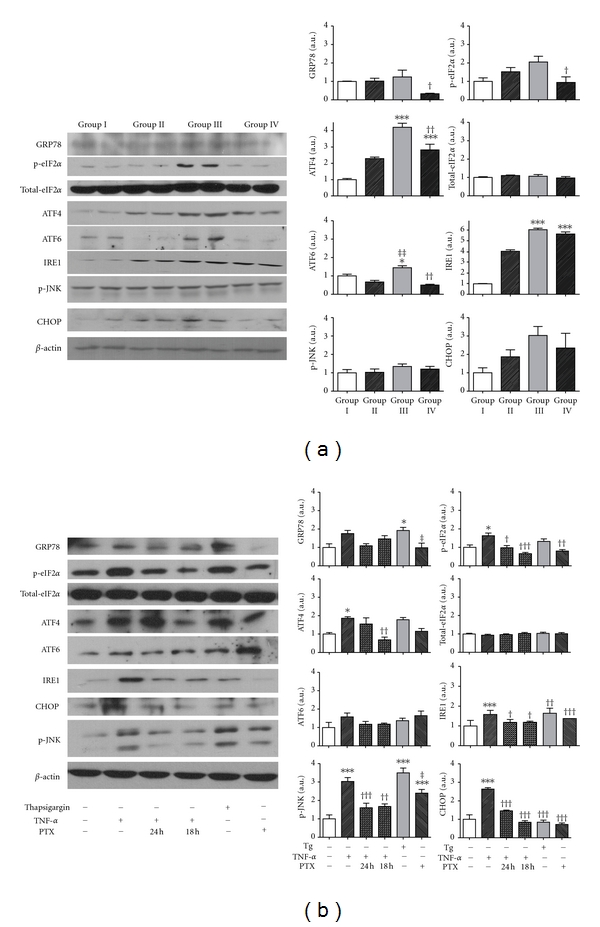
Effect of pentoxifylline on TNF-*α*-induced ER stress in Hep3B cells and hepatocytes. Rats fed MCD diet (group III) showed activated levels of GRP78, phosphor-eIF2*α*, ATF4, ATF6, IRE1, p-JNK, and CHOP with significance in ATF4, ATF6, and IRE1. Compared to group III, administration of PTX in MCD diet rats (group IV) attenuated the expression of GRP78, phosphor-eIF2*α*, ATF4, ATF6, p-JNK, and CHOP (a). The experiments were performed five times under identical conditions. Results are shown as mean ± SEM. ***P < 0.001 *versus* untreated control group. ^†^ *P* < 0.05, ^††^ *P* < 0.001: MCD plus saline *versus* MCD plus PTX group. Hep3B cells exposed to 100 ng/mL TNF-*α* for 24 h activated GRP78, p-eIF2*α*, ATF4, ATF6, IRE1, p-JNK, and CHOP. Pretreatment of 1 mM PTX for 18 and 24 h reduced TNF-*α*-induced ER stress in Hep3B cells (b). The experiments were performed four times under identical conditions.

ER stress markers were measured in liver protein levels *in vivo*. Compared to chow-diet-fed rats (group I), rats fed MCD diet (group III) showed increased levels of GRP78, phosphor-eIF2*α*, ATF4, ATF6, IRE1, p-JNK, and CHOP, with significant increases found in ATF4, ATF6, and IRE1 (*P* < 0.05 for ATF6, *P* < 0.001 for ATF4 and IRE1). Compared to MCD-diet-fed rats in group III, the expression of GRP78, phosphor-eIF2*α*, ATF4, ATF6, IRE1, p-JNK, and CHOP was attenuated in PTX-administered, MCD-fed rat (group IV), significantly in GRP78, p-eIF2*α*, ATF4, and ATF6 (*P* < 0.05 for GRP78 and p-eIF2*α*, *P* < 0.01 for ATF4 and ATF6, resp.) (Figure 5(a)).

Hep3B cells exposed to 100 ng/mL TNF-*α* for 24 h activated GRP78, phosphor-eIF2*α*, ATF4, IRE1, and CHOP *in vitro*. Significant increases were found in phosphor-eIF2*α*, ATF4, IRE1, p-JNK, and CHOP (*P* < 0.05 for p-eIF2*α*, ATF4 and *P* < 0.001 for IRE1, p-JNK, and CHOP). Pretreatment with 1 mM PTX for 18 and 24 h reduced TNF-*α*-induced ER stress in Hep3B cells, significantly in p-eIF2*α*, ATF4, IRE1, p-JNK, and CHOP (Figure 5(b)).

## 4. Discussion

Similar to unsatisfactory explanation for pathophysiology of nonalcoholic fatty liver disease (NAFLD) encompassing steatosis plus necroinflammation, controversy remains regarding the efficacy of therapeutic strategy targeting oxidative stress and TNF-*α* [4, 5] in the treatment of NAFLD. Although heterogenous results of pentoxifylline have been reported from randomized controlled trials of NAFLD treatments [4], consistent evidence in pentoxifylline-treated patients regarding improved liver aminotransferase levels, its safety of treatment for the treatment of alcohol-related liver disease, and significant improvement on mortality suggest pentoxifylline as a candidate for NAFLD treatment [6]. Few studies have investigated the effects of pentoxifylline on inflammation-induced NAFLD animal model. In view of these facts, our attentions were focused on the antihepatotoxic effect of pentoxifylline (PTX), a nonselective TNF-*α* inhibitor, on improvement of inflammation and fat droplets accumulation in methionine- and choline-deficient-(MCD-) diet-induced steatohepatitis. We hypothesized that PTX may inhibit TNF-*α*-induced endoplasmic reticulum (ER) stress and reactive oxygen species (ROS) pathway, allowing for alleviation of steatohepatitis *in vitro* and *in vivo*. Concerning animal models used to investigate the pathogenesis or treatment efficacy of steatohepatitis characterizing human condition, we adopted MCD-diet-induced rat steatohepatitis model. MCD diet depletes hepatic antioxidants, such as GSH and S-adenosylmethionine (S-AMe), and induces production of TNF-*α* and other proinflammatory cytokines [7]. According to one study, after 26 days of MCD feeding, serum ALT levels increased consistently, and steatohepatitis ultimately developed [1]. In this study, the rats were fed either chow or MCD diet for 4 weeks.

This study has two main findings. First, in accordance with previous reports, the administration of MCD diet for four weeks induced steatosis, inflammation and ballooning degeneration of hepatocytes, but not pericellular fibrosis. Endoplasmic reticulum (ER) stress was found to cause the degradation of misfolded or unfolded proteins in the ER through three pathways, including double-stranded RNA-activated protein kinase—like ER-resident kinase (PERK), inositol-requiring protein-1 (IRE), and activating transcription factor-6 (ATF6) pathwaies [8]. Similar to previous reports on ER stress in development of NAFLD [8, 9], MCD diet increased hepatic TNF-*α* expression and activated ER stress factors such as Bip/GRP78, p-eIF2, ATF4, ATF6, IRE1, and CHOP. Treatment with 100 ng/mL TNF-*αin vivo* also caused ER stress. The discrepancies of statistical significance between hepatocytes acquired from MCD diet rats and Hep3B cells on the expression of ER stress markers in the group of TNF-*α* only and the groups of both TNF-*α* and PTX may be attributed to nonuniform pattern of downstream of ER stress markers, especially ATF4, CHOP, and GADD34 in human and animal subjects with NAFLD and NASH [10].

In contrast with the human metabolic profiles of hepatic consequences of metabolic syndrome [12], MCD-fed animals demonstrate weight loss associated with atrophy of adipose tissue [2]. In this experiment, chow diet resulted in an increase in rat weight by approximately 132.9 g, but MCD diet resulted in a decrease in weight of approximately 53.6 g after four weeks of diet. This weight loss in the MCD diet group led to 50.8% and 58.3% decrease in serum triglyceride and total cholesterol level compared to the chow diet group, with statistical significance. Compared to the controls, MCD-induced steatohepatitis also led to 50.8% and 233.0% increase in AST and ALT levels, with statistical significance found only in ALT level.

Second, intraperitoneal treatment with pentoxifylline in rats fed MCD diet decreased fat accumulation. However, the effects of PTX on either body and liver weight or serum lipid profile and aminotransferases were not profound. The minimal effects of PTX on metabolic profiles, while contradictory to the expected results, may be attributed to the overwhelming effect of MCD diet due to prolonged 4-week feeding model adopted in this study. Conventional duration of the MCD diet ranges from 2 to 3 weeks [1–3]. However, compared to MCD-fed rats, rats fed PTX showed significantly decreased hepatocellular expression of TNF-*α* mRNA. *In vitro*, pretreatment with 1 mM PTX in the presence of 100 ng/mL TNF-*α* attenuated ER stress. We have suggested that pentoxifylline has anti-inflammatory effects against cytokine-induced steatohepatitis. Therefore, as we and others have found, pentoxifylline might exert its main effects through alleviation on ER stress.

To summarize our data, we observed that pentoxifylline (PTX) alleviated ER stress in TNF-*α*-induced cytotoxic human hepatocarcinoma Hep3B cells *in vitro* and had protective effect on the development of steatohepatitis in a methionine- and choline-deficient (MCD) diet animal model *in vivo*. Further clinical studies are needed to evaluate the use of PTX for nonalcoholic fatty liver disease.

## Figures and Tables

**Table 1:** Metabolic effects of MCD diet and pentoxifylline on SD rats.

Group	Chow + saline	Chow + PTX	MCD + saline	MCD + PTX
Initial BW (g)	269.2 ± 39.3	265.1 ± 37.6	268.4 ± 34.9	270.7 ± 39.0
Final BW (g)	402.1 ± 32.0	385.6 ± 33.5	214.8 ± 30.4	207.3 ± 32.1
Weight change (g)	132.9 ± 14.2	120.5 ± 10.5	−53.6 ± 9.2	−63.4 ± 10.2
Liver weight (g)	12.4 ± 1.5	11.7 ± 1.0	10.1 ± 2.8	9.8 ± 2.2
LW/BW (%)	3.1 ± 0.0	3.0 ± 0.0	4.7 ± 0.0	4.7 ± 0.0

LW: liver weight, BW: body weight.
